# Age Is Relative—Impact of Donor Age on Induced Pluripotent Stem Cell-Derived Cell Functionality

**DOI:** 10.3389/fcvm.2018.00004

**Published:** 2018-01-25

**Authors:** Elisabeth Tamara Strässler, Katriina Aalto-Setälä, Mostafa Kiamehr, Ulf Landmesser, Nicolle Kränkel

**Affiliations:** ^1^Charité – Universitätsmedizin Berlin, corporate member of Freie Universität Berlin, Humboldt-Universität zu Berlin and Berlin Institute of Health, Berlin, Germany; ^2^Partner Site Berlin, German Centre for Cardiovascular Research (DZHK), Berlin, Germany; ^3^University of Tampere, Department of Medicine and Life Sciences, Tampere, Finland; ^4^Heart Center, Tampere University Hospital, Tampere, Finland

**Keywords:** induced pluripotent stem cells, aging, senescence, cell therapy, cellular reprogramming

## Abstract

Induced pluripotent stem cells (iPSCs) avoid many of the restrictions that hamper the application of human embryonic stem cells: limited availability of source material due to legal restrictions in some countries, immunogenic rejection and ethical concerns. Also, the donor’s clinical phenotype is often known when working with iPSCs. Therefore, iPSCs seem ideal to tackle the two biggest tasks of regenerative medicine: degenerative diseases with genetic cause (e.g., Duchenne’s muscular dystrophy) and organ replacement in age-related diseases (e.g., end-stage heart or renal failure), especially in combination with recently developed gene-editing tools. In the setting of autologous transplantation in elderly patients, donor age becomes a potentially relevant factor that needs to be assessed. Here, we review and critically discuss available data pertinent to the questions: How does donor age influence the reprogramming process and iPSC functionality? Would it even be possible to reprogram senescent somatic cells? How does donor age affect iPSC differentiation into specialised cells and their functionality? We also identify research needs, which might help resolve current unknowns. Until recently, most hallmarks of ageing were attributed to an accumulation of DNA damage over time, and it was thus expected that DNA damage from a somatic cell would accumulate in iPSCs and the cells derived from them. In line with this, a decreased lifespan of cloned organisms compared with the donor was also observed in early cloning experiments. Therefore, it was questioned for a time whether iPSC derived from an old individual’s somatic cells would suffer from early senescence and, thus, may not be a viable option either for disease modelling nor future clinical applications. Instead, typical signs of cellular ageing are reverted in the process of iPSC reprogramming, and iPSCs from older donors do not show diminished differentiation potential nor do iPSC-derived cells from older donors suffer early senescence or show functional impairments when compared with those from younger donors. Thus, the data would suggest that donor age does not limit iPSC application for modelling genetic diseases nor regenerative therapies. However, open questions remain, e.g., regarding the potential tumourigenicity of iPSC-derived cells and the impact of epigenetic pattern retention.

## Introduction

Average life expectancy in most Western countries is above 80 years and still rising (1). This development poses enormous challenges to both society and health care (2), as elderly patients suffer more frequently from chronic and degenerative diseases, such as chronic heart failure or kidney failure (3). Induced pluripotent stem cells (iPSCs) might represent a source of cellular material for regenerative therapies in those conditions, thereby avoiding limitations that impede the usage of embryonic stem cells (ESCs), namely immunological problems, limited availability of source material as well as legal and ethical concerns.

Thus, ever since the first description of iPSCs (4), hopes have been high for a quick translation of the technology into clinically applicable therapeutic strategies. This hope has increased since the advent of novel gene-editing tools such as CRISPR/Cas9 technology (5).

Given the fact that the elderly are the patient group with the greatest potential benefit from iPSC-based regenerative therapies, questions regarding the impact of donor age on the functionality of iPSCs and their offspring have arisen. This review aims to give an overview of the current base of knowledge regarding those issues and identifies unanswered research questions to tackle in the future.

## iPSC Reprogramming—How does it Work and How does Donor Age Affect It?

### Cellular Reprogramming

Cellular reprogramming is achieved by overexpression of the four Yamanaka factors (Sox-2, Oct-3/4, Klf-4, and c-Myc) in somatic cells, originally done using retroviral vectors (4). Since then other methods that avoid DNA-alterations have been described, such as adenoviral vectors (6) or recombinant proteins (7). More recently, the Sendai virus, an RNA virus, has been introduced (8), which has advanced to be the most widely used tool for cellular reprogramming in recent years.

**Figure 1:**
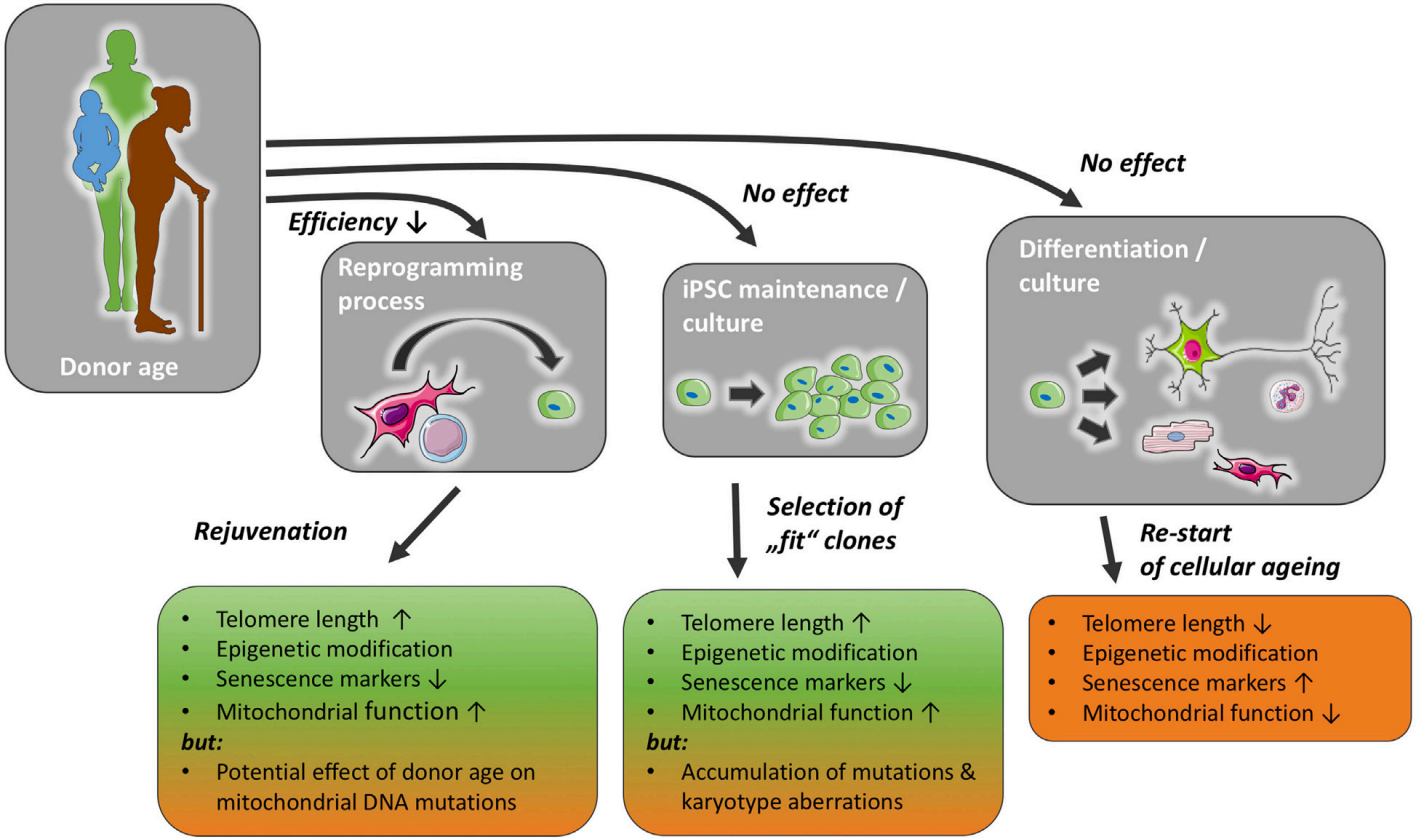
Donor age may reduce reprogramming efficiency, but has not been found to affect induced pluripotent stem cell (iPSC) maintenance or differentiation capacity, with a possible exception of the accumulation of mutations in mitochondrial and nuclear DNA. The reprogramming process and iPSC culture impact markers of cellular senescence and likely ensure a selection of functionally healthier clones. With prolonged iPSC culture, however, the risk of karyotype changes and the accumulation of mutations might increase. The normal ageing process is re-initiated once stemness mechanisms, such as telomerase function, are reduced in mature cells differentiated from iPSC.

During the process of cellular reprogramming, many internal changes occur in the cell, e.g., telomerase reactivation, changes in methylation patterns and mitochondrial morphology, as well as a decrease in senescence markers, e.g., p21 (9–12). It is thought today that iPSCs can be propagated indefinitely. In accordance with this, they retain telomerase activity and a stem cell-like epigenome, their mitochondria appear immature and are less in number compared with somatic cells (13). However, it has been shown that iPSCs can gain DNA mutations as well as karyotype aberrations (14) (Figure 1). Although it should be noted that the incidence of chromosomal aberrations is not higher than in ESCs (15). Following cellular reprogramming, iPSCs can be differentiated into somatic cells, e.g., endothelial cells, cardiomyocytes, or neurons. With the differentiation into somatic cells, the cellular ageing process restarts, although iPSC-derived somatic cells exhibit longer telomeres, improved mitochondrial function, lower senescence markers, p21 and p16, than their parent somatic cell (10, 16). Definitions of senescence and aging, as well as typical markers of senescence are discussed in Boxes 1 and 2.

Box 1 **What’s in a word? Ageing versus senescence.**

An early theory of ageing identified accumulated DNA damage as the sole culprit of this inevitable process (64). In the meantime, it has been understood that ageing most likely is a multifactorial process affecting cells on multiple levels (65). Recently, nine hallmarks of ageing have been described, these are telomere attrition, genomic instability, epigenetic alterations, mitochondrial dysfunction, cellular senescence, stem cell exhaustion, altered inter-cellular communication, deregulated nutrient-sensing, and loss of proteostasis (66); the first five being most relevant to iPSC technology. Thus, cellular senescence is only a part of ageing and describes the loss of a cell’s division capacity. Multiple internal and external stimuli can induce cellular senescence, e.g., DNA damage or telomere loss, many ultimately leading to an upregulation of cell-cycle inhibitors, such a p21 (67). Finally, ageing has been recognised as a multifactorial process involving a decrease in efficiency and function of multiple cellular maintenance mechanisms with cellular senescence being the most obvious sign in cell culture.

Box 2 **What makes the clock tick? Senescence markers.**

- **Telomere attrition**: telomeres are nucleotide repeat caps at the end of each chromosome. With each cell division, telomere length shortens due to imperfect DNA replication but also other harmful influences on the cell, e.g., oxidative stress (68). This telomere shortening is also observed in ageing, meaning cells from older individuals have shorter telomeres than those of younger ones (69).
- **Loss of mitochondrial function**: another sign of cellular ageing is mitochondrial dysfunction which is believed to be mainly due to accumulated damage caused by oxidative stress caused by prolonged close proximity of the mitochondrial DNA to reactive oxygen species as well as less efficient DNA repair mechanisms in mitochondria compared with the nucleus (70, 71).
- **Genomic instability**: during its lifespan, a cell is continuously exposed to DNA altering stimuli, e.g., ROS (72) or radiation (73), and although most damage is repaired quickly, DNA mutations accumulate over time. This is also due to a loss of DNA repair efficiency (58). Furthermore, DNA damage is also introduced by erroneous DNA replication (74) and chromosomal segregation (75, 76).
- **Histone modifications/epigenetic changes**: during a cell’s lifespan, not all genes need to be transcribed at any one time and inhibition of gene transcription is in part conveyed by DNA methylation (77). Patterns of epigenetic alterations can be attributed to different cellular characteristics, e.g., senescence-associated patterns (35) or age-associated patterns (56). This is of special importance in cellular reprogramming seeing as not all forms of epigenetic memory are influenced in kind during cellular reprogramming. It is in part this fact, which makes iPSCs such a valuable research tool; donor-specific epigenetic characteristics remain unchanged during cellular reprogramming (78) and more importantly stay unchanged in iPSC-derived somatic cells (9). Otherwise, a reliable disease modelling through iPSC-derived would not be possible seeing as it has emerged over the years that not only genetic but also epigenetic characteristics can influence and cause disease (79).

Each of these steps and the resulting cells could, in theory, be affected by the age of the donor in various ways (Figure 1).

### Effect of Donor Age on iPSC Reprogramming Efficiency

Very low reprogramming efficiencies for somatic cells from old donors compared with young ones have been reported (17) as well as the retention of age-associated DNA methylation patterns (18). In mice, it has been shown multiple times that donor age influences reprogramming efficiency (19, 20). Still, even cells from centenarians have on several occasions successfully been reprogrammed to iPSCs (10, 21), proving that advanced donor age on its own poses no insurmountable obstacle to the process of cellular reprogramming. Furthermore, multiple strategies have been found to improve cellular reprogramming efficiency, such as targeting senescence effectors (22) or easing access to pluripotency genes through C/EBPα (23). Still, it should be kept in mind that in human cells, it is up to now impossible to separate the impact of the very variable genetic background in the human population from the effect of age on iPSC reprogramming efficiency (24). Due to this variation, any comparison of interindividual reprogramming efficiency based on donor characteristics, such as age, is inherently flawed.

Another factor of equal importance for reprogramming efficiency is the time somatic cells are cultured before iPSC derivation (25). Moreover, prolonged passaging of fibroblasts was accompanied by a decrease in reprogramming efficiency and an upregulation of p21, a marker of cellular senescence (25). Thus, special care should be taken to optimise culture conditions when reprogramming cells from elderly donors.

In addition, different treatments have been shown to improve reprogramming efficiency for senescent cells, e.g., adding Nanog and LIN28 to the classic four Yamanaka factors (10) or knockdown of p21 (25). These techniques might be of special interest in donors of very advanced age where only a limited number of somatic cells for reprogramming can be collected. However, in most cases, the number of cells collected with a normally sized blood sample is more than adequate for deriving iPSCs (26). Furthermore, the addition of ZSCAN10, a pluripotency factor, has been demonstrated to normalise ROS scavenging by glutathione and DNA damage response in iPSCs from old donors, thereby improving their quality (27).

## Hallmarks of Ageing—How are they Affected by Donor Age and Cellular Reprogramming?

### Telomere Length

Early cloned organisms showed a decreased lifespan in comparison to their non-cloned counterparts (28), and it was theorised that shorter telomere length might be one reason for this observation (29). Reports of early iPSC-derived cells’ premature senescence seemed to underscore the validity of this (17). Nevertheless, it has been demonstrated that during cellular reprogramming telomere length increases through reactivation of telomerase, and that telomerase reactivation is even necessary for successful reprogramming of somatic cells into iPSCs (30, 31). Some researchers showed that telomere length does vary considerably between different iPSC lines (11) but that even iPSCs derived from centenarians have telomere lengths comparable to ESCs (10). With iPSC-derived fibroblasts still showing longer telomere length than their parent somatic cells after multiple passages (32). This strongly suggests that regarding telomere length, donor age does not negatively influence iPSC nor their somatic offspring (Figure 1).

### Mitochondrial Function

A hallmark of human ESCs is their more immature mitochondrial morphology, and indeed, iPSCs show a similar mitochondrial morphology suggesting that iPSCs are comparable to their embryonic counterparts (33). Global assessment of mitochondrial function and energy metabolism in iPSCs and ESCs further emphasises their similarities (13). This mitochondrial “de-ageing” also leads to an improved mitochondrial function in iPSC-derived cells (16). Recently, however, it has been reported that iPSCs from older individuals carry more mutations in mitochondrial DNA (34), indicating that further research into this subject is needed and no conclusion on the influence of donor age on mitochondrial function in iPSCs and iPSC-derived cells might be drawn at this time.

### Epigenetic Modifications

Unlike disease-specific epigenetic changes, age-associated histone modifications are reverted to a more embryonic-like state by the reprogramming process (35). Furthermore, prolonged cultivation of iPSCs leads to an even stronger rejuvenation, i.e., methylation patterns become increasingly similar to ESCs the longer the iPSCs remain in culture (36). Senescence-associated epigenetic patterns undergo a similar rejuvenation process during cellular reprogramming and iPSCs can be passaged multiple times *in vitro* without entering a senescent state (35). Furthermore, it has been shown that iPSCs from both young and old donors exhibit a negative calculated age (based on CpG site methylation patterns) but still iPSCs from young donors score in the lower negatives than those of old donors (18). A problem lies in the retention of tissue-specific epigenetic alterations which in part could be caused by incomplete reprogramming and might be improved by vigorous quality testing and careful selection of iPSC colonies during reprogramming and passaging (37). Retention of tissue-specific epigenetic characteristics might prove a hindrance to iPSC differentiation into specific types of somatic cells. This fact might explain the observed inter-cell line disparity in differentiation efficiency (38). Furthermore, it has been shown that endothelial-derived iPSCs can be differentiated more easily into endothelial cells than iPSCs derived from other tissues of the same individual (39). If this proves true, the tissue for sample collection could be chosen according to the later therapeutic application to improve iPSC differentiation as well as iPSC-derived cell function, e.g., sample muscle tissue if the desired tissue for later clinical use is iPSC-derived cardiac muscle. Further studies are needed to answer the question whether retention of tissue-specific DNA methylation patterns is due to incomplete reprogramming as opposed to a natural characteristic of iPSCs.

### DNA Mutations

With the preservation of genetic information during cellular reprogramming, one possible problem in using somatic cells from older individuals is the higher frequency of genetic aberrations (40). In fact, gene-disrupting DNA mutations in iPSCs increase with donor age and have been associated with cellular dysfunction and cancer (18). Another potential problem of iPSC technology is that some of the patients’ cells might undergo mutation during the reprogramming process itself (41). Repeated passaging of the iPSCs might be able to mitigate both of these problems regardless of the age of the donor, as it appears non-mutated iPSCs have a growth advantage (42) (Figure 1). However, the data have been contradictory in this regard with some researchers also reporting an increase in both genetic mutations (14) and karyotype aberrations (15) with prolonged time in culture. This certainly has to be further investigated before a large-scale clinical application of iPSC technology is possible. This issue was illustrated in 2015 when the first trial with iPSC-derived retinal pigment epithelial autologous cell transplantation had to be halted due to one patient’s iPSC-derived cells harbouring oncogene mutations (43, 44). However, it also shows that adherence to a thorough quality assessment of iPSC-derived cells before the final clinical application can prevent potential harmful effects (the trial has since been resumed; UMIN-CTR number: UMIN000011929).

## Functionality and Ageing of iPSCs and iPSC-Derived Cells—How is it Affected by Donor Age?

Induced pluripotent stem cells have to fulfil several criteria: virtually indefinite propagation capacity, the ability to give rise to cells from all three germ layers, as well as teratoma formation. None of these criteria seem to be negatively affected by donor age. Indeed, it has been demonstrated that even iPSCs of centenarians can be passaged over 110 times, can give rise to cells of all three germ layers, and are able to form teratomata (10, 21).

With regard to iPSC-derived cell function, early publications stated that iPSC-derived somatic cells show early senescence when cultured *in vitro* (32). However, this has not been confirmed (45). On the contrary, multiple hallmarks of cellular ageing (e.g., telomere attrition, age- and senescence-associated DNA methylation patterns) are reversed after the introduction of the four Yamanaka factors into somatic cells, as has been discussed before (Figure 1).

In mice, it has been shown that iPSC-derived cell functions are not negatively affected by donor age. In one study, iPSCs from both old and young mice were differentiated into vascular progenitor cells and transplanted into a mouse model of hindlimb ischaemia. No difference in iPSC differentiation efficiency was observed. In addition, both groups injected with iPSC-derived vascular progenitor cells showed comparable, improved hindlimb revascularisation compared with non-treated animals (46). Another study showed that cardiac tissue of iPSC-derived chimeric mice, with iPSCs derived from the bone marrow of 2- and 18-month-old mice, showed no difference in telomere length or mitochondrial gene expression and no upregulation of p16 nor p53 was detected between both age groups (47).

In humans, too, donor age does not seem to negatively impact on iPSC-derived cell functionality. This was demonstrated by Chang et al. who compared both iPSCs and iPSC-derived erythroid cell quality derived from cells of donors of variable age (embryonic, fetal liver mesenchymal stem cells, and adult fibroblasts) and found no difference between the age groups (48). Another study showed the same in iPSC-derived fibroblasts, where fibroblasts from both old and young women were reprogrammed to iPSCs and further differentiated into iPSC-derived fibroblasts. Both groups of iPSCs expressed comparable levels of pluripotency (Nanog, Oct-4, Sox-2, hTERT, SSEA-4, Tra-1-60, and Tra-1-81), senescence (p21 and p53), as well as apoptosis markers (Bax). Donor age did not negatively impact iPSC-derived fibroblast mitotic nor senescence-associated β-galactosidase activity (49). Indeed, when high-quality iPSC clones were chosen for further differentiation, even iPSC-derived fibroblasts from a 96-year-old donor could be cultured for 62 population doublings, signifying a 50% gain of proliferation capacity (10). Another study by Chang et al. showed that iPSC-derived erythroid cells from adult donors produced fetal and not adult haemoglobin supporting cellular rejuvenation during the reprogramming process (50). Furthermore, in a single-case study, Prigione et al. demonstrated that iPSC obtained from an 84-year-old woman were able to differentiate into all three germ layers in embryoid body formation assays (12). Still, it has to be kept in mind that available findings are derived from a very low number of iPSC lines and thus statistical as well as methodological limitations apply. Systematic studies assessing the impact of donor age on iPSC differentiation capacity in a large number of lines all produced and maintained under the same conditions are necessary to provide a final answer on the effect of donor age on iPSC differentiation capacity.

## Cellular Rejuvenation during iPSC Generation—Is It a Problem for Modelling of Late-Onset Diseases?

### Induced Pluripotent Stem Cells

One of the current key applications of iPSC technology is disease modelling, seeing as cell replacement therapy usually necessitates additional gene therapy of iPSCs before the cells or tissue might be transplanted. Moreover, in sight of the fact that iPSC-derived tissues show signs of rejuvenation, modelling of late-onset illnesses is particularly challenging (51). Nonetheless, multiple ways of circumventing this problem have been suggested recently. One way, for example, is to overexpress progerin, the protein causing Hutchinson–Gilford progeria syndrome (52); another is to shorten telomeres in the iPSC-derived cells (53). Both cause a phenotype similar to aged cells in iPSC-derived cells, enabling the modelling of late-onset diseases.

The initial difficulties in modelling late-onset diseases should be seen as another indication that iPSC-derived tissues are at least partly rejuvenated and harbour great potential for future clinical applications, especially in elderly patients.

### Nuclear Transfer

When talking about potential clinical applications of iPSC-derived cells, nuclear transfer should also be taken into consideration. After the discovery of iPSCs, nuclear transfer has taken a back seat in the field of regenerative medicine in part due to its much lower reprogramming efficiency. However, there are distinct differences inherent in the reprogramming process that might make nuclear transfer-derived stem cells (NT-SCs) a superior therapeutic option for a select number of patients.

For instance, nuclear transfer leads to longer telomeres in derived stem cells (54) and seems to erase epigenetic memory better than cellular reprogramming, for NT-SCs are more similar in their DNA methylation patterns to ESCs than iPSCs (38). However, there is some contention on this point as contradictory data have been published (55). Another important fact is that mitochondria in NT-SC derived tissues stem from the oocyte donor and not from the patient themselves. Thus, NT-SCs could help in the treatment of mitochondrial diseases, e.g., Leigh syndrome or mitochondrial myopathy.

However, production of NT-SCs is costlier and more time-consuming compared with iPSC derivation. To obtain oocytes, female donors must undergo hormonal stimulation, which carries a considerable risk for adverse effects (e.g., deep venous thrombosis or pulmonary embolism), and the harvesting process, if relatively safe, is still an invasive procedure in a person who will gain no later treatment benefit. However, seeing as mitochondrial diseases tend to manifest in young patients, there seems to be no particular benefit in nuclear transfer technology for elderly patients at the moment.

## Conclusion

In an ageing society, regenerative therapies are of great scientific and clinical interest and hold significant potential therapeutic benefits. Not only could they further prolong life but they could also—and this may be even more important—significantly improve quality of life for elderly patients suffering from chronic diseases such as diabetes, chronic heart failure, chronic kidney failure, or even neurodegenerative diseases (e.g., Parkinson’s or Alzheimer’s disease). Regenerative therapies using iPSC-derived cells, tissues, or organs are an ideal option because they avoid ethical dilemmas, immunological rejection of transplanted tissue, and other problems discussed earlier. However, before these clinical benefits can come to fruition, essential questions need to be answered.

Some regard the influence of donor age on iPSCs and iPSC-derived cell function and quality, which have been answered to some extent above. In a nutshell, iPSCs appear rejuvenated on a global scale: their telomere length is increased, mitochondrial function is improved, and epigenetic patterns are comparable to ESCs (16, 31, 33, 56). This rejuvenation is also passed on to the iPSC-derived somatic cells (10). Nonetheless, mutations in nuclear and mitochondrial DNA acquired over the donor’s lifespan and during the reprogramming process might persist. It remains to be verified if careful clone selection during iPSC culture and rigorous quality control are enough to overcome this.

As stated before, at the moment, it is not yet known how strongly the variable genetic background of individual donors affects the reprogramming process and the quality of resulting iPSCs. This certainly poses a significant obstacle to quantifying the influence of donor age on the whole process of cellular reprogramming. To more precisely pinpoint the influence of donor age on iPSCs and iPSC-derived cell function, it would be prudent to use cells from the same individual collected at different time points. For instance, it has become increasingly popular for parents to cryogenically preserve the umbilical cords of their children (57), many of which are grown up by now. Thus, it would be relatively easy to assess the impact donor age has on the above-mentioned qualities in the same individual, thereby minimising the confounding influence of interindividual genetic differences. Another point of contention is the tissue most often chosen for cellular reprogramming, namely, skin. Over a human’s lifespan, our skin is exposed to all kinds of mutagenic influences, e.g. UV radiation (58, 59). This could influence reprogramming efficiency and later iPSC-derived cell quality much more than donor age itself. Also, other locations for taking tissue samples are to be assessed critically. For instance, Wen et al. used vaginal fibroblasts as source material, but they did not screen for human papillomavirus, a prevalent infection which is known to be mutagenic and could, thus, be of importance as well (49, 60). In addition, it has to be stated that the number of cell donors was exceedingly low, namely *n* = 1 per group (49), which hardly allows for a meaningful statistical analysis. Due to the significant time and effort needed for even one iPSC line, a low number of donors and cell lines is a general problem in almost all research articles on the topic of iPSCs. This combined with the lack of a standardised protocol for optimal iPSC derivation, culture and quality control makes any comparison between different publications very difficult if not impossible. Especially, since it has been shown that many factors influence the quality of iPSCs and iPSC-derived cells, such as time and cell type used for reprogramming, time in culture, or reprogramming modality (25, 61). Particularly with the increasing number of different tissues used for iPSC derivation (e.g., skin fibroblasts, PBMCs from blood, or cells isolated from urine), further studies into the preservation of tissue-specific DNA methylation patterns as well as the influence of sample tissue on differentiation potential of iPSCs and quality are required.

Also, other important questions are still unanswered, such as potential tumourigenicity of iPSC-derived cells as exemplified by the halted trial using iPSC-derived retinal pigment epithelial cells in 2015 in Japan (43, 44). On the other hand, it also demonstrated that quality control was able to prevent the implantation of substandard iPSC-derived cells. The question regarding tumourigenicity will most likely only be answered satisfactorily once the differentiation methods are further improved, iPSC-derived cell-based therapies have made their way further into clinical practice, and patients receiving treatments have been observed for multiple years. In the meantime, animal studies could add valuable insights. For instance, in 2014, rhesus macaques were injected with undifferentiated iPSCs which lead to teratoma formation, whereas injection of iPSC-derived mesodermal stromal cells leads to bone formation without teratoma formation (62). Recently, first animal studies combining gene therapy with iPSC technology have been published with positive results. In one study, iPSCs were derived from β-thalassaemia patients, underwent gene therapy, and were then differentiated into haematopoietic stem cells. These haematopoietic stem cells were then injected into non-lethally irradiated mice. In turn, these mice then showed an improved haematopoiesis compared with those injected with non-treated iPSCs from the same patients and—maybe more importantly—no tumour growth was observed (63).

With regard to the impact of donor age on iPSCs and iPSC-derived cell quality and function, most studies point to a cellular rejuvenation during cellular reprogramming which is transferred to iPSC offspring, and the usage of somatic cells of old donors for iPSC derivation seems unproblematic, provided culture conditions are of a sufficient standard. On the contrary, iPSC-derived cells from old donors show improved functionality and, thus, seem ideal as a means of therapeutic interventions.

## Author Contributions

ES with NK performed the literature research and wrote the article. NK, MK, UL, and KA-S critically revised the paper and discussed interpretation of literature.

## Conflict of Interest Statement

The authors declare that the research was conducted in the absence of any commercial or financial relationships that could be construed as a potential conflict of interest.
